# Roles of p53-Mediated Host–Virus Interaction in Coronavirus Infection

**DOI:** 10.3390/ijms24076371

**Published:** 2023-03-28

**Authors:** Xue Wang, Yi Liu, Kaiyuan Li, Zhihui Hao

**Affiliations:** College of Veterinary Medicine, China Agricultural University, Beijing 100193, China

**Keywords:** p53, coronavirus, antiviral

## Abstract

The emergence of the SARS-CoV-2 coronavirus has garnered global attention due to its highly pathogenic nature and the resulting health crisis and economic burden. Although drugs such as Remdesivir have been considered a potential cure by targeting the virus on its RNA polymerase, the high mutation rate and unique 3’ to 5’ exonuclease with proofreading function make it challenging to develop effective anti-coronavirus drugs. As a result, there is an increasing focus on host–virus interactions because coronaviruses trigger stress responses, cell cycle changes, apoptosis, autophagy, and the dysregulation of immune function and inflammation in host cells. The p53 tumor suppressor molecule is a critical regulator of cell signaling pathways, cellular stress responses, DNA repair, and apoptosis. However, viruses can activate or inhibit p53 during viral infections to enhance viral replication and spread. Given its pivotal role in cell physiology, p53 represents a potential target for anti-coronavirus drugs. This review aims to summarize the relationship between p53 and coronaviruses from various perspectives, to shed light on potential targets for antiviral drug development and vaccine design.

## 1. Introduction

Coronaviruses (CoVs) are a diverse family of single-stranded positive-sense enveloped RNA viruses that can induce a range of issues, including the stress response of host cells, cell cycle changes, cell apoptosis, and host immune dysregulation and inflammation [1]. They can infect humans and various vertebrates, leading to severe public health problems and economic losses worldwide [2,3]. Coronaviruses were first isolated from chickens in 1937. Usually, the host range of CoVs is very narrow, and there are six coronaviruses other than the 2019 coronavirus disease (COVID-19) that can infect humans [4]. However, the multiple coronavirus pandemics that have occurred in recent years, such as the 2019 SARS-CoV-2, the 2012 middle east respiratory syndrome (MERS), and the 2002 severe acute respiratory syndrome (SARS) outbreaks, have demonstrated the potential for the zoonotic and human-to-human transmission of emerging coronaviruses [5,6]. Coronaviruses use various strategies to create an optimal environment for replication, including inducing cell cycle arrest, evading host immune proteins, and modulating cellular processes such as apoptosis and autophagy. Inducing cell cycle arrest slows down or halts the cell’s replication machinery, allowing the virus to redirect cellular resources toward viral replication [7]. Coronaviruses also employ multiple mechanisms to evade the host’s natural immune defenses, including blocking immune signaling molecules and altering the expression of host immune proteins [8]. Moreover, coronaviruses can manipulate apoptosis and autophagy by inducing or inhibiting these processes, as needed, to promote their survival and replication [9]. The recurrence of these viruses and human endemic coronaviruses indicate that future outbreaks are likely, making it essential to understand their pathogenesis and find safe and effective treatment methods.

**Figure 1:**
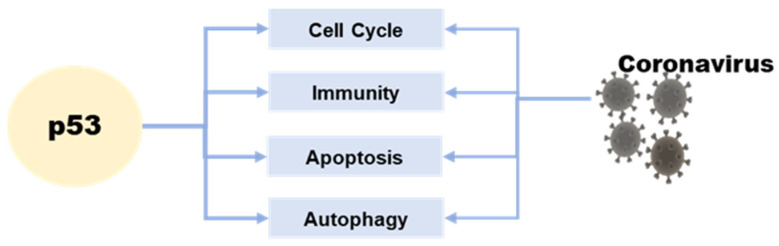
The relationship between p53 and coronavirus.

The p53 tumor suppressor is a crucial molecule attending to the regulation of various basic cell signaling, including cell cycle, DNA repair, apoptosis, and cellular stress response [10,11,12]. Moreover, apart from its role in regulating cell growth and apoptosis, p53 also plays a crucial role in various cellular processes, including metabolism, autophagy, and innate immunity [13]. This protein can directly or indirectly influence cellular metabolism, impacting the production and consumption of energy within the cell [14]. Additionally, p53 is involved in the process of autophagy, which plays a key role in removing damaged cellular components and recycling cellular building blocks [15]. In addition to its role in regulating cell growth and division, p53 also plays a role in regulating the innate immune response, such as interferon production [16]. p53 is a protein that can be found in both the nucleus and cytoplasm of a cell. In the nucleus, p53 specifically binds to DNA. Normally p53 checks for DNA damage spots in the G1 phase, monitoring the integrity of the genome [17,18]. Upon detecting DNA damage, the p53 protein activates a signaling pathway that can halt cell division and initiate DNA repair mechanisms. If the damage is severe and cannot be repaired, p53 can induce apoptosis, a process of programmed cell death. [19]. If both copies of the p53 gene are mutated, cell proliferation goes out of control, and the cell becomes cancerous [20,21]. In normal physiological mammalian cells, p53 is maintained at low levels, usually by continued ubiquitination and subsequent degradation by murine double minute-2 (MDM2) [10,22,23]. Different amounts of MDM2 can inactivate p53 in different ways. The polyubiquitination and degradation of p53 in the nucleus are caused by high levels of MDM2, and p53 monoubiquitination and nuclear exclusion are caused by low levels of MDM2 [24]. The p53 protein is involved in a negative feedback loop with MDM2, which targets p53 for destruction. Therefore, the activation of p53 is usually accompanied by an inhibition of MDM2 levels. However, when cells encounter stressors such as DNA damage, hypoxia, or viral infection, p53 ubiquitylation is inhibited, allowing it to accumulate in the nucleus. In this location, p53 undergoes multiple covalent modifications, including phosphorylation and acetylation, which activate and stabilize the protein [25]. p53 is intricately linked to viral infection, with both beneficial and detrimental effects. This review summarizes the relationship between p53 and coronavirus development, providing insights into treating human and animal coronaviruses (Figure 1).

## 2. p53 Affects Coronaviruses by Regulating the Cell Cycle

Cell cycle progression may be an advanced and orderly regulated method. Throughout this method, multiple units work alongside to make sure of smart division and proliferation [26]. Cyclin-dependent kinases (CDK) and cyclins mediate the cell cycle progression [27]. Inhibition or absence of CDK activity can result in cells’ arrest in the G1 phase and their entry into a quiescent state [28]. The G1-S and G2-M phases of the cell cycle provide protection against both exogenous and endogenous agents that can cause DNA damage [29]. The molecular level changes in these two stages are complex and susceptible to environmental conditions caused by coronaviruses [30]. P21 is an inhibitor of CDKs and regulates the cell cycle by interfering with cyclins [31]. The transition from the S phase to the G1 phase is facilitated by cyclins. CDKs are inhibited by p21WAF1/CIP1, which causes low phosphorylation of retinoblastoma (Rb) and prevents E2F release, thereby blocking the transformation of the G1-S and G2-M phases. G2-M arrest is mainly regulated by the p53-p21-DREAM pathway, which indirectly inhibits the transcription of cell cycle genes such as CCNB2, KIF23, and PLK4 [32]. In addition to inducing G2 arrest through 14-3-3 stimulation that sequesters cyclin B1-CDK1 complexes outside the nucleus, p53 can also cause cell cycle arrest by transactivating the GADD45 and 14-3-3 genes. These are among the various functions of p53 [30].

**Figure 2:**
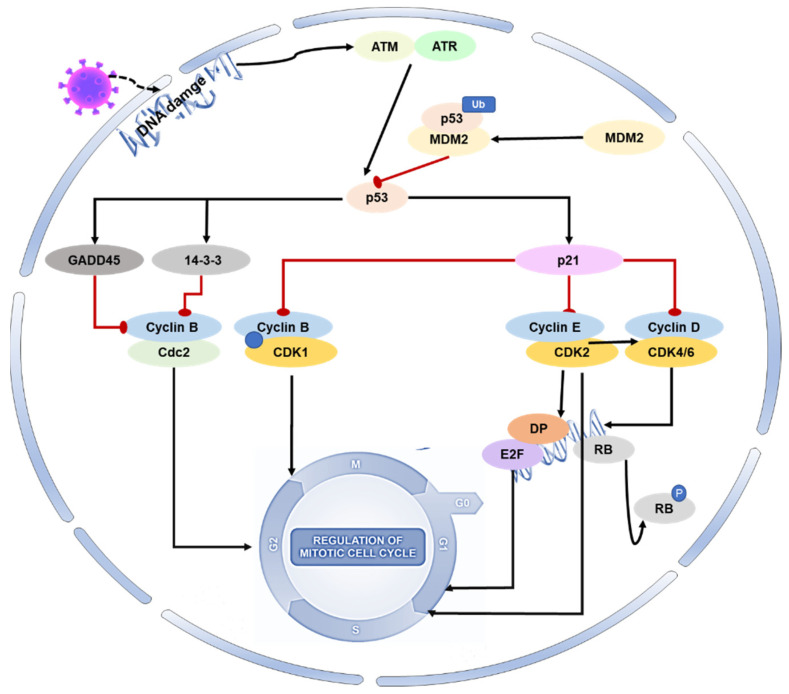
Role of p53 on the cell cycle during coronavirus infection. During viral infection, p53 is activated and initiates a cascade of events that result in cell cycle arrest. Specifically, activated p53 mediates the protein p21 to form complexes with CDKs, resulting in the inhibition of cyclin–CDK activity. This also leads to a reduction in Rb phosphorylation, thereby maintaining the stability of the RB–E2F complex and inhibiting the transcription of cell cycle genes. Additionally, p53 mediates the proteins 14-3-3 and GADD45 to inhibit cyclin–CDK1 activity, resulting in cell arrest in the G2-M phase.

Regulation of the host cell cycle could be a common strategy several viruses use for infectious agent replication (creating an excellent cellular environment). Coronavirus infection not only induces oxidative stress and DNA damage in host cells, but it can also interfere with the host cell cycle by directly altering the activity of p53 or its upstream and downstream proteins [33,34,35,36,37]. SARS-CoV-2 N protein can induce acute kidney injury by arresting the cell’s G1 cycle [38]. With the deepening of coronavirus–host interaction research, the p53-DREAM pathway provides a novel antiviral strategy. Porcine epidemic diarrhea virus (PEDV) N protein induces S-phase arrest in host cells by activating p53 and binding downstream protein DREAM to CHR [39]. Mouse hepatitis virus (MHV) infection reduces G1 cyclin–CDK complexes and induces G0-G1 cell cycle arrest [40]. Infectious bronchitis virus (IBV) induces G2-M phase perturbation in both asynchronous and synchronous replication cells, thereby inducing G2-M phase cell cycle arrest to promote progeny virus replication and reproduction [41]. Research has shown that the SARS coronavirus can hinder the activity of cyclin–CDK complexes, leading to reduced phosphorylation of retinoblastoma protein and decreased E2F1-mediated transcriptional activation. This, in turn, can impede S-phase progression in mammalian cells [42]. Infection with PEDV can modify the expression of proteins involved in cell cycle regulation, such as p21, CDK1, CDK2, CDK4, cyclin A, and cyclin E. The viral infection can cause cell cycle arrest in the G0-G1 phase, which can be restored by inhibiting the p53 signaling pathway. This leads to the downregulation of p21 and associated cyclin/CDK proteins [43] (Figure 2).

## 3. The Interaction between p53 and Interferon

IFNs possess diverse biological activities, which include antiviral effects, antiproliferative effects, and the activation of immune cell cytotoxicity. IFNs are central to antiviral immunity and can inhibit coronaviruses’ replication. Cells produce type I IFN (primarily IFN-α and IFN-β) in response to viral infections, which is critical for immunity against many types of viruses. The transcription of antiviral-related genes is induced by IFN-I, which can be triggered by viral recognition sensors such as toll-like receptors and RNA helicases such as the RNA helicase retinoic acid-inducible gene I (RIG-I). The activation of interferon regulatory factor (IRF) leads to the production of type I interferon [44]. IFNAR1 and IFNAR2 form a type I IFN receptor that recognizes and binds to type I IFN, giving bystander cells antiviral effects. The phosphorylation of STAT-1, STAT-2, and IRF9 transcription factors is increased by the activation of the JAK-STAT signaling pathway. These factors then form a heterotrimeric complex called IFN-stimulated gene factor 3 (ISGF3) and translocate to the nucleus [45]. This complex has been shown to activate p53 transcription but is not associated with p53 phosphorylation [46].

p53 also contributes to the increased release of IFN-1 from virus-infected cells [47]. IRF9 was confirmed to be a p53 target gene, suggesting that type I IFN can not only increase the expression of p53 by activating IRF9 but also that p53 can activate IRF9. IRF9 continues to activate retinoic acid inducer 1 (RIG-I) and ISRE-dependent genes such as IRF7 [47,48]. IRF3 and IRF7 play a crucial role in inducing the expression of type I interferon genes downstream of pattern recognition receptors. These transcription factors bind to the promoters of IFN-α and IFN-β through homologous or heterologous interactions, thereby controlling their expression [49]. In addition, recent studies have revealed the relationship between p53 and the cGAS/STING innate immune system pathway. p53 induces the ubiquitination of three prime repair exonuclease 1 (TREX1) through the ubiquitin ligase TRIM24. The degradation of TREX1 prevents the timely removal of cytoplasmic DNA, thus activating the cGAS/STING pathway and increasing the synthesis of IFN [50].

**Figure 3:**
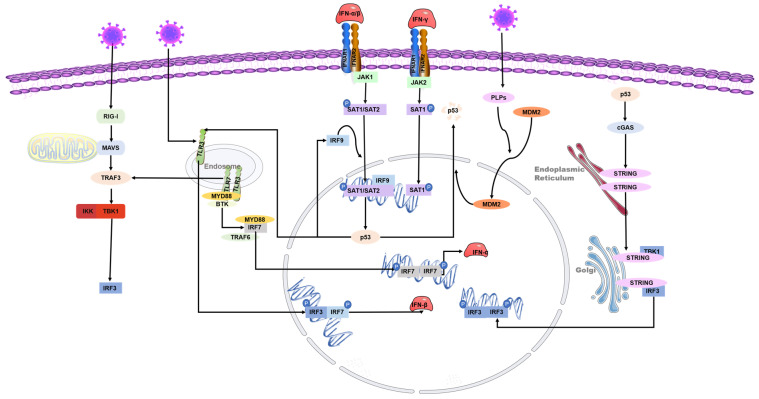
Role of p53 in the regulation of interferon during coronavirus infection. The toll-like receptor and RNA helicase retinoic acid-inducing gene I (RIG-I) are activated after the RNA virus invasion of cells. IRF3 is phosphorylated to produce type I interferon. The JAK-STAT signaling pathway is subsequently activated, and the transcription factors STAT-1, STAT-2, and IFN regulatory factor 9 (IRF9) bind to induce p53 expression. IRF9 is also a p53 target gene and is regulated by p53. Virus infection activates p53-mediated TLR3, phosphorylates downstream IRF3 and IRF7 to form dimers, and promotes the synthesis of type I interferon. p53 also activates IRF3 through the p53-cgas-STING pathway. In addition, coronaviruses can evade the host’s natural immune defenses by degrading p53 through their papain-like proteases (PLPs).

p53 can inhibit the replication of coronaviruses and shows antiviral activity in vivo; this effect may be due to its ability to activate natural immune pathways. Previous studies have shown that several coronaviruses, such as SARS-CoV-2, SAR-CoV, and human coronavirus NL63 infections, can only induce deficient levels of IFN-I [51,52,53], which is likely to lead to uninhibited viral replication and damage to the immune system. Low-level IFN responses may be a means by which coronaviruses evade immunity. The lack of adequate IFN response may be due to the decrease of p53 hydrolyzed by the coronavirus papain-like proteases (PLPs). PLPs are a class of cysteine proteases that inhibit innate immunity by stabilizing the binding of MDM2 and p53 to cause the ubiquitination of p53 [54]. The SARS-unique domain (SUD) and papain-like proteases (PLPs) interact with cell E3 ubiquitin ligase and CHY zinc-finger domain-containing 1 (RCHY1) to facilitate their activities. SUD and PLPs target p53 with E3 ubiquitin ligase RCHY1 to degrade p53. The degradation of p53 decreases the level of earthly IFN [55]. In other words, coronaviruses can evade the host’s natural immune defenses by degrading p53 through their own proteins. At the same time, the application of small molecule inhibitors of MDM2, such as nutlin-3 and idasanutlin, can promote the stable presence of p53 in cells, help regulate the IFN signaling pathway, and inhibit the replication of coronaviruses [56] (Figure 3).

Interferon is a common clinical strategy for treating coronavirus infection [57]. In the case of MERS-CoV, delayed treatment with IFN-β not only failed to effectively inhibit virus replication but also increased the expression of pro-inflammatory cytokines, resulting in mouse mortality [58]. In addition, delayed IFN-I treatment promoted SARS-CoV infection and caused severe acute pneumonia in SARS-CoV-infected mice [59]. Recent studies have shown that IFN treatment reduces epithelial cell proliferation and differentiation, exacerbating COVID-19 disease and susceptibility to bacterial co-infection [53]. Therefore, the use of IFN in treating various coronavirus infections should be considered with caution due to time and other issues.

## 4. p53 Affects Coronavirus-Associated Apoptosis

**Figure 4:**
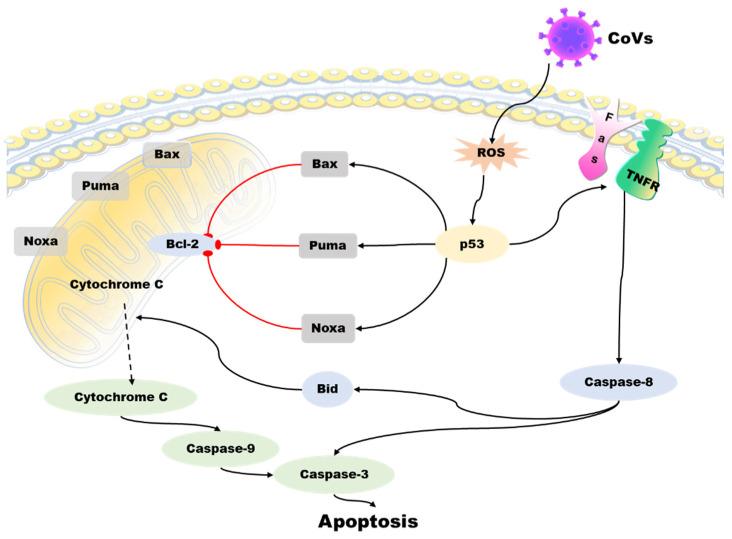
Role of p53 in the regulation of apoptosis during coronavirus infection. The virus induces ROS accumulation in cells and activates p53 protein, which activates the pro-apoptotic proteins of the Bcl-2 family -Bax, Noxa, PUMA, and BID, antagonizing the anti-apoptotic effect of BCL-2. Then, cytochrome C from mitochondria spills over, triggering a cascade of caspase proteins, leading to apoptosis. p53 can also act on the tumor necrosis factor receptor (TNFR) family to activate caspase-3 and caspase-8 through the exogenous apoptotic pathway and cause apoptosis.

Apoptosis is a programmed death mechanism evolved by cells in response to the complex external environment and self-injury. This mechanism is dangerous but effective for living organisms. Due to the particularity and importance of apoptosis, there is a complicated regulatory mechanism network, and p53 is one of the links. Apoptosis is primarily controlled by intrinsic and extrinsic pathways. The intrinsic pathway of apoptosis involves a complex downstream signaling network that is regulated by p53 [60]. The TNFR family, including the death receptor and Fas, initiates the extrinsic pathway of apoptosis by activating the formation of the death-inducing signaling complex (DISC). This leads to the activation of caspases, including caspase-8 and caspase-3, ultimately resulting in apoptosis [61]. The Bcl-2 family of proteins regulates the intrinsic apoptotic pathway, and it includes anti-apoptotic and pro-apoptotic members. Anti-apoptotic proteins, such as Bcl-XL, can perform anti-apoptotic functions due to their structural similarity to Bcl-2. In contrast, pro-apoptotic proteins such as Bax and Bak can antagonize the anti-apoptotic function of Bcl-2 because they share a similar structure with Bcl-2 and Bcl-XL. The external apoptotic pathway involves the activation of caspases, including caspase-8 and caspase-3, through the death-inducing signaling complex (DISC) formation initiated by the tumor necrosis factor receptor (TNFR) family (such as death receptor and Fas), leading to apoptosis. P53 plays a crucial role in regulating the intrinsic pathway of apoptosis [62]. Interestingly, Bax, Noxa, PUMA (p53-up-regulated apoptosis regulator), and BH3 interaction domain death agonists (BID) are important targets for p53. p53 can upregulate the expression level of these proteins, thus inducing apoptosis [63]. Other studies have shown that p53 can directly stimulate mitochondria to release high ROS and cause cell apoptosis [64] (Figure 4).

In many traditional views, virus-induced apoptosis is regarded as an antiviral strategy for the host. The relationship between apoptosis and viruses is very subtle and complex. For some viruses, inhibiting host cell apoptosis can increase the replication time in the cell, and rapid cell death may reduce the replication level. The human cytomegalovirus (HCMV)–encoded UL37 exon-1 protein (UL37 × 1) can increase HCMV replication and infection by inhibiting host cell apoptosis proteins. Meanwhile, the immunosuppressive and anti-apoptotic activity of UL37 × 1 is essential for HCMV replication in vivo [65]. Flaviviruses, such as the Zika virus (ZIKV), produce subgenomic flavivirus RNA (sfRNA) using host mRNA degradation mechanisms. sfRNA can modulate the expression of genes involved in cell death pathways in the mosquito genome, leading to the inhibition of cell apoptosis in mosquito tissue. This process can prolong viral genome replication and promote virion assembly, which, in turn, facilitates viral infection [66]. Hepatitis C virus (HCV) NS5A proteins form complexes with inositol triphosphate receptor type 3 (IP3R3) and F-box and leucine 2 (FBXL2), promoting the FBXL2-mediated degradation of IP3R3. Degradation of IP3R3 inhibits calcium flux, reduces mitochondrial calcium overload, and leads to apoptosis. Therefore, the anti-apoptotic effect of NS5A prolongs the replication time of HCV and is conducive to HCV infection [67]. However, in some cases, activation of the apoptotic pathway can promote viral replication and spread. The ORF3a protein of SARS-CoV-2 has been shown to induce apoptosis in infected cells [68]. Porcine deltacoronavirus (PDCoV) infection triggers the release of apoptotic cells into the cytoplasm by stimulating mitochondrial outer membrane permeabilization (MOMP) via either Bax recruitment or mitochondrial permeability transition pore (MPTP) opening. This results in the activation of an intrinsic caspase-dependent apoptotic pathway that promotes viral replication in vitro [69]. PEDV induces apoptosis in Vero cells through a series of events that include increasing intracellular ROS accumulation, which, in turn, increases MDM2 and CBP expression. This process further stimulates the phosphorylation of p53 at serine 20, promotes the nuclear translocation of p53, and activates p53, ultimately leading to apoptosis [64]. Because of the inextricable link between coronavirus infection and apoptosis, p53 is a link that cannot be bypassed.

## 5. p53 Affects Coronavirus-Associated Autophagy

Autophagy is a process by which cells recycle cellular components and eliminate damaged organelles or misfolded proteins. It is also a critical mechanism for the innate immune response against viral infection. p53 regulates autophagy through transcription-dependent and transcription-independent mechanisms. It can stimulate autophagy by upregulating genes such as damage-regulated autophagy modulator (DRAM) and sestrin2. Additionally, p53 can interact directly with autophagy-related proteins, such as LC3 [70,71]. However, p53 can also inhibit autophagy by upregulating genes such as tuberous sclerosis complex 2 (TSC2) and PTEN, which negatively regulate the mTOR pathway, a key regulator of autophagy [72].

However, some viruses, including coronaviruses, can hijack the autophagy pathway to promote their own replication and spread. p53 has a dual role in regulating autophagy during coronavirus infection. On the one hand, it can stimulate autophagy as a host defense mechanism against viral infection [73]. For instance, p53 can induce autophagy in response to SARS-CoV-2 infection, leading to the degradation of viral particles and the suppression of viral replication. Similarly, p53-mediated autophagy inhibits the replication of other coronaviruses, such as HCoV-229E and MERS-CoV [74]. On the other hand, some coronaviruses can manipulate the p53 pathway to promote their own replication by inhibiting autophagy. For instance, the SARS-CoV-2 virus downregulates p53 expression and induces MDM2 expression. This leads to the degradation of p53, which inhibits p53-mediated autophagy and promotes viral replication [55,75]. Similarly, the MERS-CoV virus also inhibits autophagy by downregulating the expression of p53 [76].

**Figure 5:**
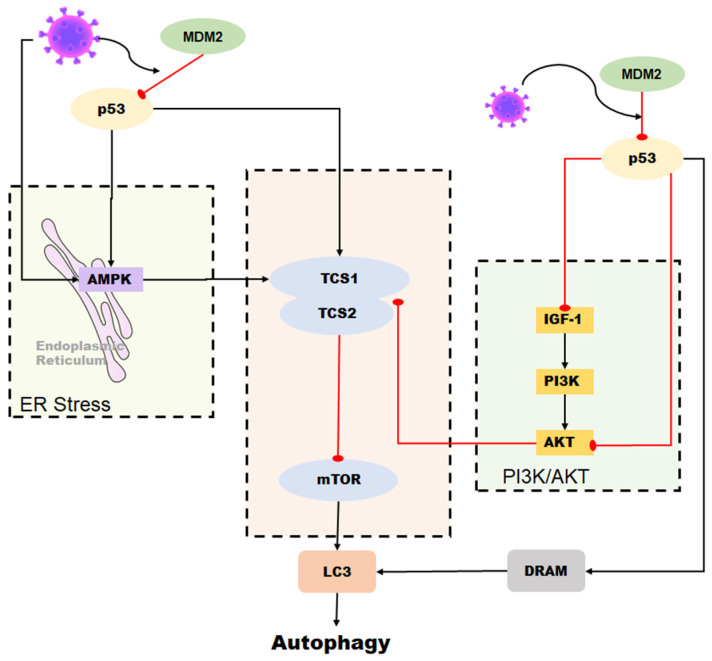
Role of p53 in the regulation of autophagy during coronavirus infection. Autophagy can be induced by p53 through the DRAM protein. In some coronaviruses, autophagy is activated by p53-mediated DRAM protein as well as through the AMPK pathway and the PI3K/AKT pathway. However, some coronaviruses can reduce the stability of p53 in cells and inhibit p53-mediated autophagy by upregulating the expression of MDM2.

In conclusion, the relationship between p53 and autophagy during coronavirus infection is complex and context-dependent. While p53 can promote autophagy as a host defense mechanism against viral infection, some coronaviruses can also manipulate the p53 pathway to inhibit autophagy and promote their own replication (Figure 5). Further research is necessary to fully comprehend the interplay between p53 and autophagy during coronavirus infection and identify potential targets for therapeutic intervention.

## 6. Coronavirus Proteins Interact with p53

Through long evolution, coronaviruses induce cell cycle arrest, apoptosis, autophagy in the invasion into host cells, genome replication, the assembly and release of virions, and immune escape [77,78,79,80]. Four major proteins, nucleocapsid protein (N), membrane protein (M), envelope protein (E), and spike protein (S), make up coronavirus particles and are called structural proteins. [81]. These proteins help coronaviruses complete their viral cycle. The coronavirus N protein regulates viral replication and transcription and plays a vital role in the viral integration of genomic RNA into progeny virions [82]. The M and E proteins and membranes from host organelles form the virion envelope. The S protein is embedded in the envelope. The function of the coronavirus highly glycosylated S protein is to help the virus adhere, enter, fuse the virus envelope to the cell membrane, and neutralize antibodies [83]. In addition to structural genes encoding structural proteins, two-thirds of the genes in the coronavirus genome encode nonstructural proteins, measuring about 20 kb [77]. RNA-dependent RNA polymerase (RDRP) is composed of nonstructural proteins expressed by these nonstructural genes; coronaviruses use RDRP complexes for genome replication and gene transcription [84].

While these proteins play their functions, p53 also becomes an important participant. Upregulation of p53 expression induced by transmissible gastroenteritis virus (TGEV) nucleocapsid protein leads to cell cycle arrest and apoptosis. Additionally, TGEV N protein induces changes in the expression of CDK1, CDK2, and cyclin B2, as well as cytochrome c translocation and Bax activation. However, inhibition of p53 can reverse these expression changes and weaken the TGEV N-protein-induced cell cycle arrest and apoptosis [85]. A previous study revealed key domains of PEDV N protein interaction with p53. PEDV N protein binding to p53 in the nucleus consistently maintains high p53 expression, thus activating the p53-dream pathway and subsequently inducing S-phase stagnation, creating a comfortable intracellular environment for viral replication [39]. Nonstructural proteins of coronaviruses have also been shown to interact with p53. RCHY1 is an E3 ubiquitin ligase that mediates proteasome degradation of its target protein. Its targets include the tumor protein p53 [86]. The papain-like proteases of SARS-CoV and MERS-CoV bind to E3 ubiquitin ligase and the RCHY1 of cells to activate and enhance RCHY1-mediated ubiquitination degradation of p53 [55]. The mouse coronavirus nonstructural protein p28 prevents the degradation of p53, and p21Cip1 is transcriptionally activated in a p53-dependent manner in cells. Increased expression of p21Cip1 inhibits cyclin E/Cdk2 activity and thus inhibits Rb hyperphosphorylation. The accumulation of Rb prevents the cell cycle from progressing from the G0-G1 phase to the S phase [87].

## 7. p53 Is a Potential Target for Anti-Coronavirus Drugs

Effective anti-coronavirus drugs rely on identifying potential targets for antiviral drugs, which are often aimed at inhibiting key viral replication proteins [88,89]. Nevertheless, RNA virus replication usually has a high error rate (or low viral fidelity), leading to viruses existing in distinct populations of genomic mutants or “quasispecies”, which means viruses can quickly develop resistance to drugs or vaccines [90]. In addition, coronaviruses have a unique 3’ to 5’ exonuclease proofreading function to remove misincorporated nucleotides from the 3’ terminus of nascent RNA, rendering most drugs targeting RNA-dependent RNA polymerase (RDRP) ineffective [91,92,93].

In recent years, more and more researchers have focused on the host cell pathway and virus–host interaction [94,95,96]. Tp53 is a crucial tumor suppressor gene in host cells. Its gene and p53 protein, encoded by Tp53, are widespread antitumor drug targets. p53-targeting therapies include but are not limited to compounds that restore/reactivate wild-type p53 function or eliminate mutant p53 [97,98,99,100]. However, p53 is not a common target in antiviral drug development. With the deepening of research on p53 in antitumor drugs, researchers have also observed its potential for antiviral effect [54,101,102]. During viral infection, p53 is activated by post-translational modifications (such as phosphorylation, acetylation, prime acylation, and methylation) with the participation of many cofactors. Activated p53 triggers a cascade that regulates the transcription and expression of its downstream targets. p53 mediates downstream targets to regulate cell cycle arrest, metabolism, apoptosis, iron death, autophagy, stem cell differentiation, DNA repair, and senescence [47,103,104,105].

First, p53 has been shown to be a target of action for many coronaviruses. Coronaviruses act with p53 to alter a range of physiological activities in host cells that are used to create a suitable environment for their own survival. The S protein, N protein, PLP protein, and RNA polymerase of coronaviruses can all interact with p53 [39,106,107,108]. Thus, p53 can be used as a potential drug target. By blocking the interaction between p53 and the virus proteins, a drug may prevent the virus from manipulating the host cells and spreading. Therefore, targeting p53 could be a promising strategy for developing antiviral drugs against coronaviruses.

In addition, if the p53-mediated biochemical response or signaling is interfered with by viral infection, reducing or eliminating this interference may be an effective antiviral strategy. This has been demonstrated in several studies. An extract from guava and lemon (including green leaf and flower) guava flavonoid glycosides (GFGs) has been demonstrated to inhibit IAV replication by the inhibition of Akt kinase reactivation of p53 during early infection [109]. Aspirin significantly reduced the level of ROS and downregulated the expression of p53 and p21, thereby inhibiting the activity of cyclin D1-CDK4 and cyclin E1-CDK2 and blocking the G0/G1 phase arrest of IAV infection significantly [110]. In the infection process of pseudorabies virus (PRV), p53 plays a potent role in RV replication and pathogenesis both in vitro and in vivo, and the infection of PRV can be effectively inhibited in p53 knockout cell lines or treated with p53 inhibitors [111]. P53 has been shown to promote high levels of human cytomegalovirus (HCMV) replication and infectious virion production in permissive cells. The decrease in p53 protein expression can inhibit HCMV infection [55]. p53 as a target of anti-coronavirus drugs has also been studied in the context of the global epidemic of SARS-CoV-2 [96]. Coronavirus papain-like protein 2 (PLP2) reduces the stability of p53 by increasing MDM53-mediated ubiquitination. This declines the p53-mediated generation of type I IFN and inhibits apoptosis, creating a cellular environment conducive to viral replication. Increased ubiquitination levels of p53 can increase human coronavirus (HCoV) infection [107]. Nutlin-3, an inhibitor of MDM2, can inhibit SARS-CoV-2 infection in the eyes by inhibiting MDM2 activity and upregulating the p53 protein level [112]. Ergosterol peroxide, a natural mushroom extract, could decrease oxidative stress in PEDV-infected cells, reduce ROS accumulation in cells, and inhibit p53 activation [113]. Triacetyl resveratrol (TCRV) is a resveratrol derivative found naturally in plants. TCRV has a good anti-PEDV effect and significantly reduces the p-p53 in PEDV-infected cells, suggesting p53 may be a drug target for TCRV [114].

## 8. Conclusions

In conclusion, despite not being considered a primary target for developing anti-coronavirus drugs, p53 presents a promising avenue for therapeutic interventions against coronaviruses. The multifaceted role of p53 in regulating cellular responses to stress and DNA damage makes it an essential player in maintaining host homeostasis, and its intricate interactions with coronaviruses highlight its potential as a critical drug target. Furthermore, recent advancements in understanding the molecular mechanisms underlying p53-mediated signaling have provided valuable insights into the development of small molecule inhibitors that selectively target p53 activity, offering a potential avenue for the development of novel anti-coronavirus therapeutics. Thus, exploring the therapeutic potential of p53 in the context of coronavirus infection presents a compelling opportunity to combat the ongoing global health crisis caused by COVID-19 and future emerging viral diseases.

## Data Availability

Data are contained within the article. The data presented in this study are available on request from the corresponding author.
